# Myopia and Inflammation

**Published:** 2011-10

**Authors:** Carl P. Herbort, Marina Papadia, Piergiorgio Neri

**Affiliations:** 1 Inflammatory and Retinal Eye Diseases, Center for Ophthalmic Specialized Care, Lausanne, Switzerland; 2 University of Lausanne, Lausanne, Switzerland; 3 Eye Clinic, Department of Neurosciences, Ophthalmology and Genetics, University of Genova, Genova, Italy; 4 The Eye Clinic, Polytechnic University of Marche, Ancona, Italy

**Keywords:** Myopia, Inflammation, Choriocapillaritis, Multifocal Choroiditis, Multiple Evanescent White Dot Syndrome, Vogt-Koyanagi-Harada Disease

## Abstract

The correlation between myopia and intraocular inflammation has rarely been explored. The aim of this article is to review myopic changes induced by inflammatory diseases and inflammatory diseases related to myopia, followed by a discussion on inflammatory choroidal neovascularization. Clinical cases are used to illustrate these conditions. The review does not include inflammatory conditions caused by surgical interventions employed for treatment of myopia. Uveitic conditions that can induce a myopic shift include sclero-choroidal inflammation, lens induced myopia due to steroid cataracts, juvenile idiopathic arthritis (JIA) induced myopia, and transient drug induced myopia due to sulfonamides and acetazolamide used for treatment of ocular toxoplasmosis and inflammatory cystoid macular edema, respectively. Most inflammatory conditions related to myopia are conditions involving the choriocapillaris. These include multifocal choroiditis and/or punctate inner choroiditis, multiple evanescent white dot syndrome and acute idiopathic blind spot enlargement. It can be hypothesized that fragility of the choriocapillaris due to particular anatomic changes due to myopia, together with unknown immunogenetic factors predispose myopic eyes to primary inflammatory choriocapillaropathies.

## INTRODUCTION

The correlation between myopia and inflammation can be subdivided into three major situations. The first category is particular consequences of inflammation which develop in highly myopic eyes; the second is myopia related to intraocular inflammation due to changes in the lens-iris diaphragm or as a side effect of therapy, including cataracts induced by corticosteroid therapy and drug induced myopic shift; the third category consists of conditions in which myopia is linked to the development of certain inflammatory eye diseases including multifocal choroiditis and its sub-entity, punctate inner choroiditis, as well as multiple evanescent white dot syndrome and acute idiopathic blind spot enlargement. Inflammatory eye diseases related to myopia have a high propensity to cause inflammatory choroidal neovascularisation (CNV), a condition that will be further discussed thereafter.

## CONSEQUENCES OF INFLAMMATION IN HIGH MYOPIA

Extreme caution should be exercised with corticosteroid therapy for uveitis in highly myopic eyes. It is well known that high myopia predisposes to glaucoma and visual field loss.^1^,^2^,^3^ These eyes are more susceptible to pressure increase following the administration of corticosteroids, the so-called steroid response.^4^ In addition to pressure increase, further myopization can occur due to decreased resistance of the thinned sclera in highly myopic eyes. These facts mandate close follow-up for highly myopic patients receiving corticosteroids in any form for treatment of uveitis. Routes of administration which can be terminated promptly, such topical or systemic corticosteroids, are preferred to periocular or subtenon injections of depot steroids.

## MYOPIC SHIFT INDUCED BY UVEITIS

Uveitis can cause acute, transient or constitutive myopization in different contexts. These include acute inflammation such as in choroidal or scleral inflammation, chronic uveitis as in juvenile idiopathic arthritis (JIA) related uveitis, as a consequence of therapy such as lens related myopization following long-term corticosteroid therapy and the use of sulfonamides for treatment of toxoplasmic retinochoroiditis or acetazolamide for cystoid macular edema (CME).

### Acute myopia due to sclero-choroidal inflammation

Severe inflammation in posterior scleritis can cause acute myopization in some cases.^5^ The possible mechanism is supraciliary exudation causing relaxation of zonular fibers and increased convexity of the crystalline lens. This phenomenon is also noted with severe choroidal inflammation such as in hyperacute Vogt-Koyanagi-Harada (VKH) disease that frequently causes acute myopia due to ciliary body detachment and inflammatory supraciliary exudation which can be detected by ultrasound biomicroscopy (UBM).^6^,^7^

#### Illustrative case of VKH disease

A twenty-year-old female patient consulted her ophthalmologist for bilateral blurred vision since several days ago. She reported flu-like symptoms and headache in the previous week. Examination showed bilateral myopic shift to −1.75 despite the fact that the patient had always been emmetropic. Best corrected visual acuity (BCVA) was 20/20 in both eyes with appropriate correction. Anterior segment examination revealed faint anterior chamber (AC) flare and rare cells in both eyes. Rare occasional cells were also noted in both vitreous cavities. Fundus examination disclosed wrinkling of the vitreoretinal interface as well as disc hyperemia in both eyes. The treating ophthalmologist was advised to perform dual fluorescein (FA) and indocyanine green angiography (ICGA). FA showed early non-homogeneous filling of the choriocapillaris with areas of delayed filling in both eyes. (Fig. 1A) On late frames, disc hyperfluorescence was noted as well as some areas of faint mottled retinal hyperfluorescence (Fig. 1B). ICGA demonstrated inflammation of choroidal vessels (Fig. 1C) as well as small regularly sized hypofluorescent dark dots evenly distributed in the posterior pole and mid-periphery (Fig. 1D). A diagnosis of VKH disease was posed and we suggested that the patient be referred for immediate treatment with intravenous corticosteroids. Unfortunately, the patient was sent home over the weekend to be seen again three days later for further investigations. At this time, she became hypermetropic by 2.0 diopters in both eyes. Repeat FA showed vast areas of subretinal pooling. On ICGA, exudative retinal detachments were seen as hypofluorescent geographic areas while regular small hypofluorescent dark dots were still present (Fig. 1E). UBM showed supraciliary exudation (Fig. 1F) explaining the myopization that had occurred before exudative retinal detachments reversed the condition to hypermetropia.

### Lens induced myopia due to steroid cataracts

A longitudinal study has shown that after corticosteroid therapy in uveitis, development of corticosteroid lens changes is accompanied by a marked increase in lens induced myopia up to more than 7 diopters due to the optical effect of the posterior subcapsular cataract.^8^

### Myopia induced by JIA-related uveitis

The relationship between refractive errors and JIA uveitis was analyzed in 65 adult patients.^9^ Refractive data were obtained at an average of 26.4 years after the onset of JIA uveitis, a disease developing in early childhood. Significantly more myopic refractive errors were found in JIA patients when compared to an age-matched control group. About 43% of patients had increased negative refractive error suggesting an association between myopia and JIA. A possible explanation for these findings is weakening of scleral connective tissue due to chronic inflammation predisposing to myopization.

### Transient drug-induced myopia

Several treatment regimens used in toxoplasmic retinochoroiditis contain agents from the sulfonamide family which may cause transient myopia.^10^ UBM explains this mechanism by demonstrating supraciliary choroidal effusion causing forward displacement of the ciliary body-lens-iris block and relaxation of zonular fibers leading to myopia, anterior chamber shallowing and angle closure glaucoma.^11^ The same mechanism can also occur when acetazolamide is used to treat inflammatory CME, since this agent also belongs to the sulfonamide family.^12^,^13^ Discontinuation of these medications leads to complete resolution of this anatomic shift. Recently, several reports of a similar condition have been published following the use of topiramate, a sulfonamide derivative used for treatment of migraine headaches, seizures, alcohol abuse and other drug dependencies.^14^

## INFLAMMATORY EYE DISEASE LINKED WITH MYOPIA

### Multifocal choroiditis

Multifocal choroiditis (MFC) is a recurrent bilateral chorioretinal inflammatory disease. Some authors further subdivide this entity into punctuate inner choroidopathy (PIC), a subset of MFC which usually occurs in young myopic patients, often involving the more myopic eye. The entity known as presumed ocular histoplasmosis syndrome (POHS) should also be included under the same group since its clinical and ICGA features are indistinguishable from MFC and both conditions probably represent the same clinical entity. Separation of these conditions is probably not warranted since their pathophysiology is the same, i.e. choriocapillaris perfusion irregularities or nonperfusion.^15^ MFC was clearly associated with myopia with an average refractive error of −2.19 diopters in a study analyzing the whole group of inflammatory choriocapillaropathies (formerly white dot syndromes).^16^

Symptoms, as in other inflammatory choriocapillaropathies, usually include photopsia, scotomata and loss of visual acuity as well as visual field defects. Fundus examination reveals small faintly visible active foci and older clearly visible yellow chorioretinal scars. Vitreous cells can be seen in the posterior vitreous during active disease.

The most useful complementary investigation is ICGA which shows two types of lesions, one are hypofluorescent dots seen in early, intermediate and late angiographic phases indicating chorioretinal scars that correspond to late hyperfluorescent non- diffusing areas on FA. During active disease, ICGA shows additional hypofluorescent areas, not apparent or barely visible as faint late hyperfluorescent areas on FA, indicating choriocapillaris non-perfusion.^17^–^19^ These ICGA lesions are reversible and usually respond to systemic or subtenon corticosteroids or triamcinolone injections.

Additional investigations should include visual field testing as in all other inflammatory choriocapillaropathies, which will show scotomata in areas with active disease and an enlarged blind spot; findings that are more pronounced than fundus lesions. It is important to exclude other causes of uveitis, especially infectious conditions such as tuberculosis.

A special feature of multifocal choroiditis is the propensity of the disease to become complicated by subretinal inflammatory CNV which is present in up to 30%–40% of cases, a proportion much higher than any other inflammatory choriocapillaropathy.

Management, as for other entities under this group, is empirical. Although there are no controlled studies, clinical experience is probably sufficient to recommend corticosteroid therapy in cases with active disease; this can usually be diagnosed when patients complain of photopsia and is further evidenced by ICGA. If corticosteroids are insufficient, immunosuppressive agents may be added. The best follow-up parameter is ICGA which can show resolution of hypofluorescent areas. If inflammatory subretinal CNV is present, corticosteroids (subtenon injections or systemic) should be tried first with concomitant or subsequent intravitreal anti-vascular endothelial growth factor (VEGF) therapy.

The outcome of MFC is often favorable if recurrences are detected early (using ICGA) and treated appropriately. The course is deleterious if follow-up is inadequate or if central lesions occur.

#### Illustrative case of MFC

A 30-year-old myopic female patient who had experienced several episodes of ocular symptoms including photopsia and scotomata since the age of 25 consulted her ophthalmologist. Retrospectively, these were considered to be the first episodes of MFC. At presentation, she complained of photopsia and subjective scotomata in her left eye. Refractive error was −10.50–1.75@26º and −9.50–1.25@158º in the right and left eyes, and BCVA was 20/25 and 20/20 respectively. There was no anterior segment inflammation and laser flare photometry values were within normal limits. There were rare vitreous cells in the left eye. Fundus examination revealed multiple foci that seemed atrophic and accounted for previous episodes of MFC (Fig. 2A). On ICGA, dark dots corresponding to chorioretinal scars were seen in the right eye. In the left eye, in addition to the same dark dots, there was a widespread geographic area of hypofluorescence indicating extensive choriocapillaris non-perfusion (Fig. 2B) which was not detectable on FA (Fig. 2C).

A course of systemic prednisolone was started at 50 mg per day and tapered over a period of 4 months. This treatment course was probably too short since several months later, a CNV developed in the right macula concomitant with recurrence of MFC in the same eye, very well evident on ICGA which revealed choriocapillaris non-perfusion; dark dots representing old cicatricial foci were also present (Fig. 2D). Corticosteroid therapy was resumed at this stage; as anti-VEGF agents were not available at the time, three courses of photodynamic therapy were performed leading to cicatrization of the CNV (Fig. 2E). Nowadays, more aggressive and prolonged immunosuppressive therapy is employed in such cases.

### Punctate inner choroidopathy

Punctate inner choroidopathy (PIC) is a subset of MFC characterized by a similar clinical picture as far as symptoms, fundus signs and neovascular complications are concerned, except that the lesions are smaller. In the original description by Watzke and colleagues, the disease was reported to be bilateral and predominant in myopic women.^17^

In an extensive report on MFC and PIC, the latter was found to be bilateral in more than 80% of cases.^20^ In the experience of some of us, the disease tends to be unilateral predominantly involving the more myopic of fellow eyes.^15^ Like multifocal choroiditis, in our hands, new lesions respond to systemic or subtenon corticosteroids and additional immunosuppressive therapy is not always necessary. Corticosteroids are also thought to have a beneficial effect on the neovascular membrane and are the first line of treatment.

#### Illustrative case of PIC

A 26-year-old female patient presented with photopsia in her left eye. Refractive error was −10.50–1.0@22º and −11.50–1.50@142º in the right and left eyes respectively with BCVA of 20/20 in both eyes. The anterior segment had no signs of inflammation but there were scarce vitreous cells in the left eye. Fundus examination was within normal limits in the right eye but on the left side, there were a few cicatricial foci in the posterior pole (Fig. 3A). Octopus visual field (VF) was normal in the right eye but showed several confluent central scotomata in the left eye (Fig. 3B). FA was within normal limits on the right side but showed several late hyperfluorescent spots indicating window-defects, ICGA showed patchy hypofluorescence extending beyond the hyperfluorescent areas seen on FA (Fig. 3C, top images). A diagnosis of multifocal choroiditis (PIC type) in the left eye was made. Systemic corticosteroid treatment was initiated starting at dose of 60 mg per day, tapered over a period of 6 months. After one month of treatment, FA remained unchanged, but ICGA revealed a decrease in hypo-fluorescent areas (Fig. 3C, middle images) parallel with an improvement in VF (Fig. 3B). After 4 months of corticosteroid therapy, all new lesions seen on ICGA regressed leaving behind only dark areas corresponding to old cicatricial atrophic areas seen on FA (Fig. 3C, bottom images). At 6 months, corticosteroids were discontinued and no recurrence was noted over 11 years of follow-up. It is noteworthy that PIC developed in the more myopic eye. Without ICGA, the recurrence would not have been detected and healing under corticosteroids could only be monitored by ICGA. On FA, no more cicatricial lesions appeared indicating that the new lesions responded well to therapy and did not lead to additional scars. The term “ICGA submarine effect” may be used under circumstances as in this case, when lesions detectable by ICGA are absolutely not visible on FA and/or fundus examination.

### Multiple evanescent white dot syndrome

Multiple evanescent white dot syndrome (MEWDS) is characterized by faint yellowish-white fundus lesions, slight papillary swelling, and moderate to severe drop in visual acuity as well as visual field defects. It is usually unilateral, limited to one episode and predominant in middle aged women but can be seen at any age.^21^ MEWDS is clearly associated with myopia as shown in at least two studies.^16^,^22^ Disease characteristics have been thoroughly described in the first two articles reporting this condition.^23^,^24^ The authors localized the functional impairment to the level of the outer retina but failed to identify the mechanism of disease. Today, it is clear that MEWDS definitively belongs to the group of primary inflammatory choriocapillaropathies (PICCPs).^25^,^26^

Symptoms include a viral flu-like prodrome, often two weeks preceding ocular symptoms, in up to 50–60% of cases; other symptoms typically include photopsia and scotomata. There is moderate to severe drop in visual acuity and visual field defects in over 90% of cases. Fundus findings include discrete yellow-white discolorations up to the mid-periphery in acute disease, and a granular appearance in the macula during the subacute phase. In some cases, an enlarged blind spot is detected and this subgroup is termed acute idiopathic blind spot enlargement (AIBSE) because other findings typical of MEWDS are not present.

The most useful diagnostic procedure is ICGA which shows hypofluorescent dots and peripapillary hypofluorescence in the acute phase which resolve over 4–8 weeks. FA shows discrete hyperfluorescent foci present on early frames or moderate disc hyperfluorescence; however FA may reveal no significant. Additional investigations may include full-field ERG which is abnormal in up to 80% of cases indicating widespread ischemia in the outer retina. The course of the disease is characterized by spontaneous resolution of signs and symptoms 6–10 weeks in nearly all cases.

#### Illustrative case of MEWDS

A 33-year-old female patient consulted her ophthalmologist 2 weeks after sensing floaters in her left eye. She also complained of a veil in front of her left eye and reported photopsia in same eye. The major symptom however, was the sensation of dark spots that tended to increase. Refractive error was −1.25–0.50@8º and −0.75–0.50@180º in the right and left eyes with BCVA of 20/20 in both eyes. Both anterior segments were non-inflamed and flare measured by laser photometry was within normal limits in both eyes. No inflammatory cells were present in the vitreous of both eyes. A slightly nonhomogeneous texture was noted in the left macula (Fig. 4A). Optical coherence tomography was within normal limits in both eyes.

Octopus visual field was normal in the right eye but showed several relative scotomata in the left one with a slight increase in mean defect (2.6 dB versus 0.1 dB in the unaffected right eye) (Fig. 4B). FA showed faint and tiny areas of hyperfluorescence and a leaking disc on late FA frames. On ICGA, geographic zones of hypofluorescence in the posterior pole were clearly identified, more clearly so in the late frames (Fig. 4C). This is a typical situation illustrating the “iceberg effect” on ICGA which is characterized by relative absence of signs on fundus examination and FA despite clear and widespread ICGA lesions. Fundus autofluorescence (FAF) was increased corresponding to hypofluorescent areas seen on ICGA; this regressed in the convalescent phase (Fig. 4D). Symptoms and visual field recovered within 7 weeks (Fig. 4B).

### Acute idiopathic blind spot enlargement

Acute idiopathic blind spot enlargement (AIBSE) was first described in 1988 in a report including 7 patients who presented with peripapillary scotomata leading to symptomatic enlargement of the blind spot confirmed by visual field testing. All patients were young, 25 to 39 years of age, with 5/2 female/male predominance.^27^ Visual acuity, color vision, pupillary responses, funduscopy and FA were all normal. The only abnormal finding was an abnormal focal ERG indicating retinal dysfunction around the optic disc at the origin of the visual field defect. Most probably, AIBSE and MEWDS are the same disease entity; the only difference is that fundus abnormalities in MEWDS are not present in AIBSE. As indicated by Hamed et al, the retinal lesions may already have subsided at the time of examination or may have been subclinical.^28^ If ICGA had been available and performed by the authors of this and subsequent reports, it is probable that AIBSE would never have been described as a separate clinical entity. ICGA is currently the method of choice for making a diagnosis of atypical MEWDS cases in patients presenting at later stages or with subclinical disease.^16^ Numerous reports on blind spot enlargement in many of the diseases presently reclassified under PICCPs indicate the presence of common physiopathogenic mechanisms.^29^,^30^ Furthermore, reports including ICGA show that visual field alterations are related to peripapillary hypofluorescence indicating choriocapillaris non-perfusion as the physiopathogenic process causing blind spot enlargement. This pathologic characteristic, found in many of the PICCPs, may indicate susceptibility of the peripapillary choriocapillaris to occlusion following inflammation leading to outer retinal ischemia and dysfunction, producing a peripapillary scotoma.

## INFLAMMATORY CHOROIDAL NEOVASCULARIZATION

### The role of inflammatory factors in CNV

A comprehensive work on inflammatory neovascularization has recently been published by P. Neri and the concepts presented herein are largely based on this work.^31^ Neoangiogenesis can occur in the presence or absence of evident inflammatory activity or can occur as a late complication of chronic low-grade inflammation. Inflammation can be subtle and choroidal lesions can often be undetectable. Choroidal inflammation is often missed unless ICGA is employed which should be considered mandatory for this group of disorders.^18^ This concept has been proven in animal models, including posterior experimental uveitis which features CNV as a late sequel.^32^–^34^ Surgically removed inflammatory CNVs have shown, on histological examination, the presence of inflammatory cells such as macrophages and lymphocytes in a higher proportion as compared to specimens obtained from eyes with age related macular degeneration (ARMD).^35^

Unfortunately, there is lack of histological studies on inflammatory CNV in humans as surgical removal is becoming less frequent with the development of efficient non-surgical treatments for CNV. The use of animal models has provided some information on the role of cells and biological mediators in the pathogenesis of CNV. Macrophage recruitment to the choroid seems to be central in the pathogenesis of CNV: Espinosa-Heidmann et al^36^ conducted a study to determine whether treatment with clodronate liposomes (CL2MDP-lip), which causes depletion of blood monocytes and lymph node macrophages, diminishes the severity of neovascularisation in a mouse model of laser-induced CNV. Recently, Jost et al^37^ proved the crucial role of plasminogen activator inhibitor-1 (PAI-1) in neoangiogenesis; the authors stated that balance between serine proteases and PAI-1 was critical for pathological angiogenesis. The large number of pathways involved in the pathogenesis of CNV and the peculiarity of the immune system within the eye make this process difficult to understand.

### The crucial role of ICGA in inflammatory CNV: the “iceberg” and “submarine” effects

Both MFC/PIC and MEWDS are classified under PICCPs, formerly called the “white dot syndromes”. Their diagnosis relies on certain ICG angiographic signs seen in this category of diseases.^18^,^19^,^38^ In inflammatory choriocapillaropathies, ICGA signs in the acute phase of the disease include irregular patchy or geographical hypofluorescent areas of variable size present in early, intermediate and late frames. (Figures 2B, 3C and 4C) This sign indicates choriocapillaris non-perfusion or hypoperfusion and is usually more clearly visible on late frames (after 28–35 seconds) after partial washout of ICG from the choroid. Very often, peripapillary ICG hypofluorescence is identified. In the convalescent phase, most of these hypofluorescent lesions regress. If ICGA hypofluorescent areas persist in the post-acute phase, they represent choroidal atrophy and scarring, changes that never occur in MEWDS.

FA is less consistent and less helpful in choriocapillaritis. Early phase FA frames, in the acute stage of the disease and parallel with ICGA, show early hypofluorescent areas indicating choriocapillaris non perfusion but only complementary ICGA can differentiate between perfusion delay or actual non-perfusion, as FA gives no more information on the choriocapillaris later than 60–70 seconds after fluorescein infusion.

In MEWDS, faint hyperfluorescence can be seen fairly early. Late fluorescein frames in the acute phase of the disease show hyperfluorescent areas going from faint to profuse hyperfluorescence depending on the severity of ischemia in the outer retina. In MEWDS, as in MFC, FA findings can be very faint (Fig. 4C) or even absent. We speak of the “iceberg effect” when ICGA findings are out of proportion to what fundus examination and/or FA reveal. The term “submarine effect” is used when ICGA choroidal lesions are completely non-detectable by both funduscopy and fluorescein angiography.

### Ischemic factors in inflammatory CNV

In PICCPs, there are double stimuli which promote the development of CNV. In addition to inflammatory factors that can trigger CNV alone such as in VKH, ischemia is always present in choriocapillaritis and adds to the inflammatory stimulus. Since each stimulus by itself can trigger CNV, this is a situation in which there is maximal danger for CNV formation. In choriocapillaris closure, ischemia is present at the level of the RPE but also more importantly in the outer retina which explains visual field dysfunction. When ischemia is very severe, it provokes “rescue” dilatation of inner retinal vessels accounting for late fluorescein staining and pooling that can be seen in severe cases of MFC and is classically seen in acute multifocal ischemic choroiditis (AMIC), also called APMPPE (acute posterior multifocal placoid pigment epitheliopathy) which is a misnomer since the primary process is not at the level of the pigment epithelium. In some PICCPs such as MFC, choriocapillaris non-perfusion is very frequent and extensive, when searched for by ICGA, explaining the high rate of CNV. In other entities such as MEWDS, ischemia is less important and less widespread decreasing, but not eliminating the risk of CNV. Very discrete choriocapillaris involvement, not detectable by any means other than ICG, can nevertheless trigger CNV^39^,^40^ and these cases probably account for the nonentity of idiopathic CNV because ICGA is still not routinely performed in these situations in a great number of ICGA refractory countries.

### Treatment of inflammatory CNV

The treatment of inflammatory CNV is still a challenge, since no guidelines are available. Treatment modalities have not been tested in controlled trials and are only based on case reports or case series at best.

Laser photocoagulation^41^, periocular and systemic steroids^42^, PDT^43^,^44^, immune suppression^45^ and surgical removal^46^ have been employed for management of inflammatory CNV in the pre anti-VEGF era.^47^

The inflammatory process leading to inflammatory CNV is not only regional, in fact the whole immune system seems to be involved.^48^ For such reasons, systemic steroids should always be considered as the primary approach to treat choroidal neovascular membranes.

The safety and efficacy of immune suppression^45^ for control of CNV in uveitis has been described. Cyclosporine A^49^ and other calcineurin inhibitors can also be used for treatment; mycophenolate mofetil (MMF) may offer a more favorable safety and efficacy profile and is a promising drug for long-term control of inflammatory CNV as recently described.^50^

As soon as PDT was introduced, argon laser photocoagulation was limited to treatment of extrafoveal neovascular membranes in order to reduce the risk of iatrogenic damage. Different strategies have been proposed for PDT; some cases have been treated electively with corticosteroid/immunosuppressive therapy and PDT^43^, or PDT was used only in case other approaches such as inflammation suppressive therapies had failed.^44^ A this time, anti-VEGF agents play a prominent role in the management of inflammatory CNV^51^,^52^ and have marginalized the role of PDT as well as that of surgery; CNV surgical removal is indicated only for extensive peripapillary membranes.^53^

Although different treatments have not been compared “head to head”, the standard of care nowadays consists of corticosteroid/ immunosuppressive treatment as the first choice for juxta/sub-foveal CNV, almost invariably administered in combination with intravitreal anti-VEGF agents.

## CONCLUSIONS

To the best of our knowledge, the relationship between myopia and inflammatory ocular diseases and its reverse, myopization in relation to inflammation, have not been studied in the last 30 years. In 1976, a report entitled “myopia and uveitis” was published in a Romanian journal.^54^ It is noteworthy to realize that acute inflammation of the sclera such as scleritis and diseases of the choroid such as VKH disease can induce myopization when the disease is hyperacute. Sulfonamides used in the treatment of toxoplasmosis and sulfonamide derived drugs such as acetazolamide employed in inflammatory CME may rarely lead to forward shift of the ciliary body-lens-iris diaphragm, and cause myopization and angle closure glaucoma. On the other hand, myopia predisposes to several ocular inflammatory conditions such as MFC and MEWDS. It should be noted that both of these entities are PICCPs. It may be hypothesized that structural globe changes due to myopia cause choriocapillaris/retinal pigmentary epithelium complex fragility, rendering it prone to inflammatory diseases.

## Figures and Tables

**Figure 1A:**
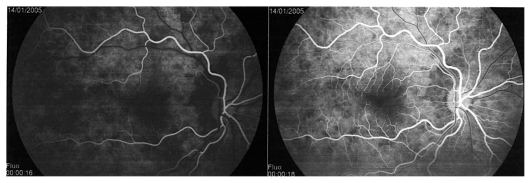
Hyperacute VKH, nonhomogeneous filling of the choriocapillaris.

**Figure 1B:**
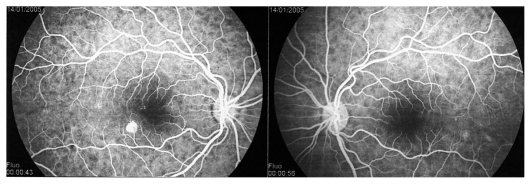
Hyperacute VKH, patchy hyperfluorescence on FA.

**Figure 1C:**
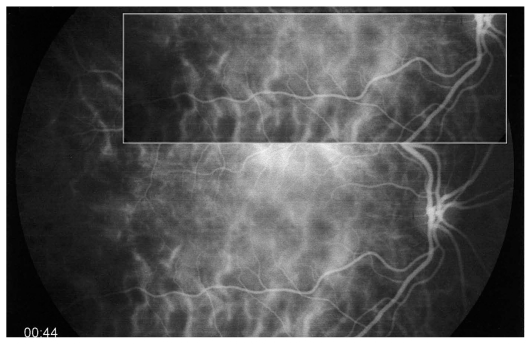
Hyperacute VKH disease, early hyperfluorescence of choroidal vessels and leakage from choroidal stromal vessels indicating choroidal vasculitis.

**Figure 1D:**
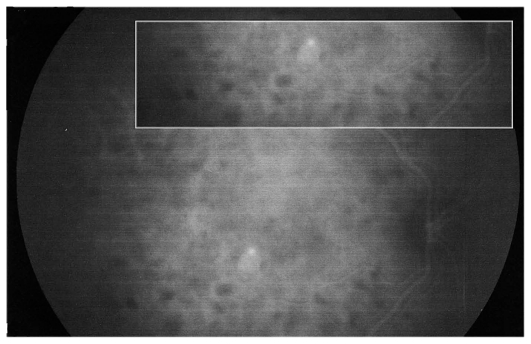
Hyperacute VKH disease, note numerous regularly sized and evenly distributed hypofluorescent dark dots on a background of diffuse choroidal hyperfluorescence, indicating granulomas of the choroid.

**Figure 1E:**
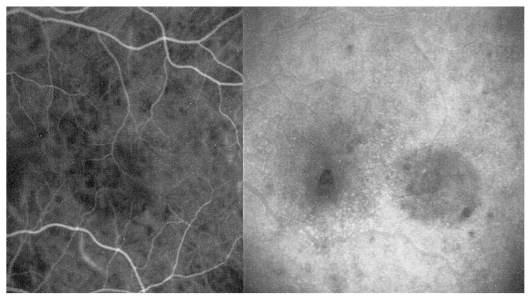
Hyperacute VKH disease, exudative retinal detachments shown on ICGA in the left eye. Note the numerous dark dots indicating choroidal granulomas.

**Figure 1F:**
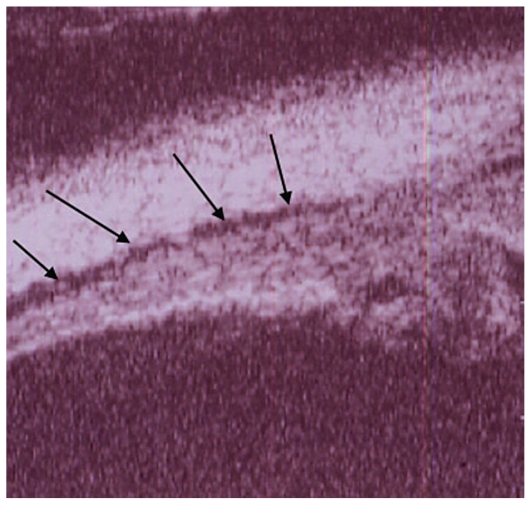
Hyperacute VKH disease, UBM image shows supraciliary effusion explaining myopization.

**Figure 2A:**
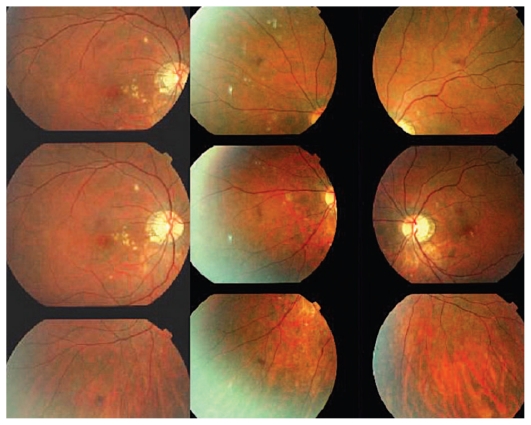
Fundus appearance in multifocal choroiditis (MFC). Right and left fundus frames of a case of MFC showing typical dispersed lesions. No sign of disease recurrence appears in the left eye.

**Figure 2B:**
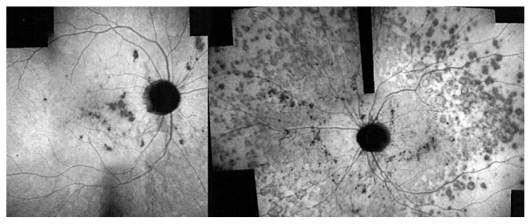
ICGA in multifocal choroiditis. ICGA shows widespread non-perfusion in the left eye not apparent on fundus photographs (Fig. 2A) nor on FA (Fig. 2C). Only ICGA allows detection of recurrence, an example of ICGA “iceberg” and “submarine” concepts indicating that ICGA signs can be pronounced despite minimal or absent fundus or FA signs. Note also the extensive ischemia which explains the high percentage of choroidal neovascularization in MFC. Right eye is currently inactive. (Courtesy of Alessandro Mantovani, Como, Italy).

**Figure 2C:**
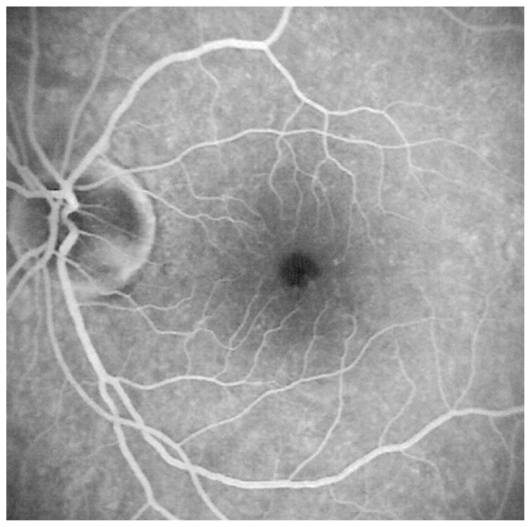
FA in multifocal choroiditis. As shown in figure 2B, extensive ICGA signs can be present in the absence of fundus or FA signs.

**Figure 2D:**
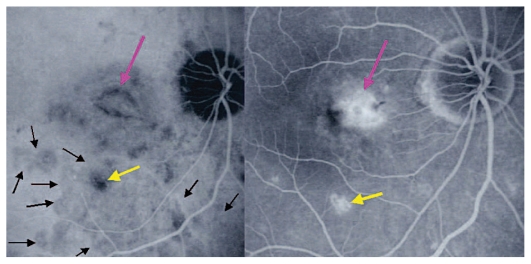
Choroidal neovascularization complicating MFC shown on ICGA (left) and FA (right). As shown in figure 2B, choriocapillaris ischemia is usually extensive in MFC and can only be detected by ICGA. This figure shows the development of CNV in the right eye (crimson arrow) concomitant with an acute recurrence of MFC causing extensive choriocapillaris ischemia (black arrows) not visible on FA. The darker areas are old cicatricial foci that can also be seen on FA (window defects, yellow arrows). (Courtesy of Alessandro Mantovani, Como, Italy)

**Figure 2E:**
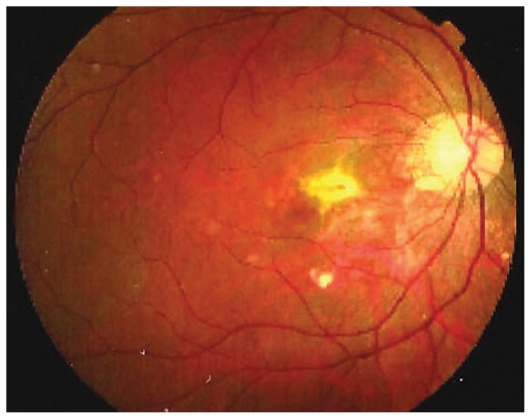
Cicatricial CNV treated by photodynamic therapy in MFC. The old cicatricial lesions that were seen on ICGA (very dark spots) and FA (window defect hyperfluorescence) are well identified.

**Figure 3A:**
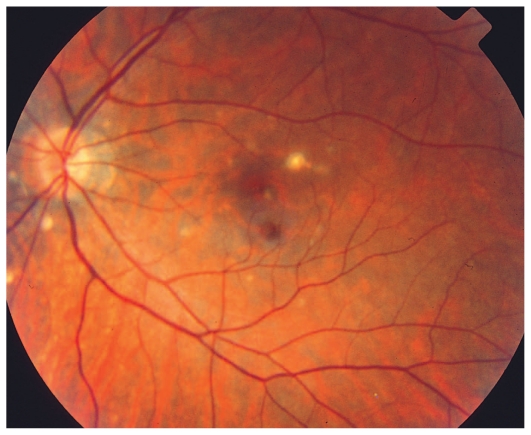
Punctate inner choroiditis, fundus image of the left eye shows one larger chorioretinal scar and several faint choroiditis foci. New recurrent lesions are not detectable.

**Figure 3B:**
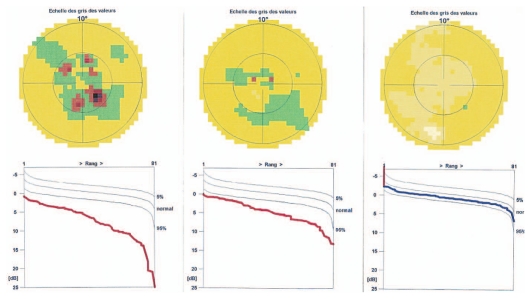
Octopus visual field at presentation and during follow-up. Extensive centrally coalescent scotomata at presentation (left). Significant improvement after one month of corticosteroid therapy (middle) and complete recovery after six months of treatment (right). (Source of illustration: reference 18)

**Figure 3C:**
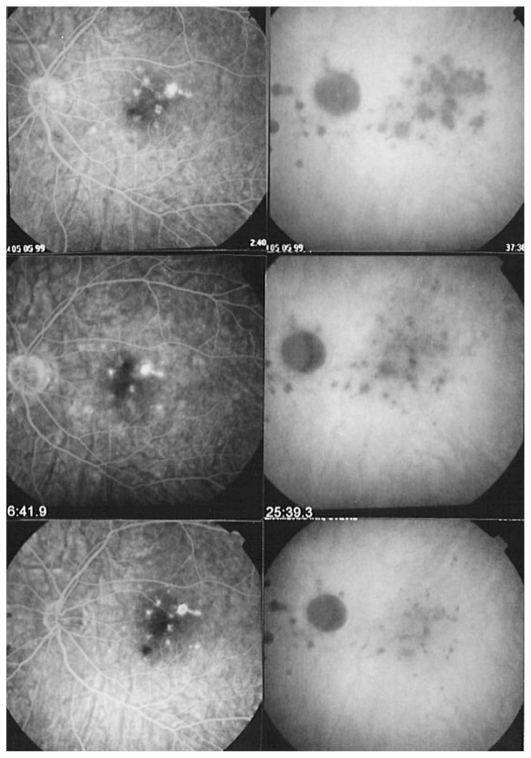
Evolution of FA (left column) and ICGA lesions (right column) at presentation and under corticosteroid treatment for punctate inner choroidopathy. At presentation (top images), FA only shows cicatricial lesions from old foci (window defects) and does not reveal the extensive occult recurrence shown by ICGA. After one month of systemic therapy, FA frames show the same cicatricial lesions (middle left) whereas the fresh lesions have regressed on ICGA (middle right). After 6 months of corticosteroid therapy (bottom images), FA shows the same appearance of old cicatricial lesions whereas ICGA shows complete regression of the fresh lesions; old atrophic scars due to episodes are also visible on FA frames.

**Figure 4A:**
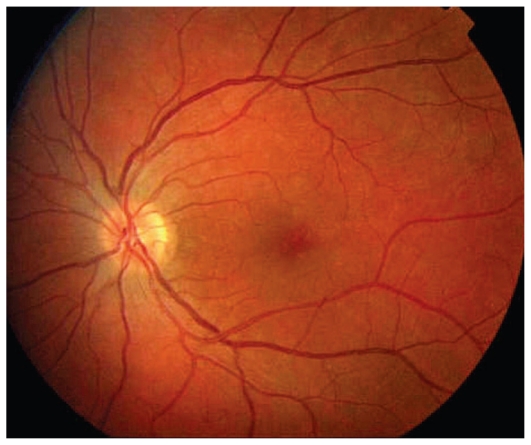
Fundus image of the left eye in a patient with MEWDS. The fundus is almost normal with only a slightly nonhomogeneous appearance in the fovea.

**Figure 4B:**
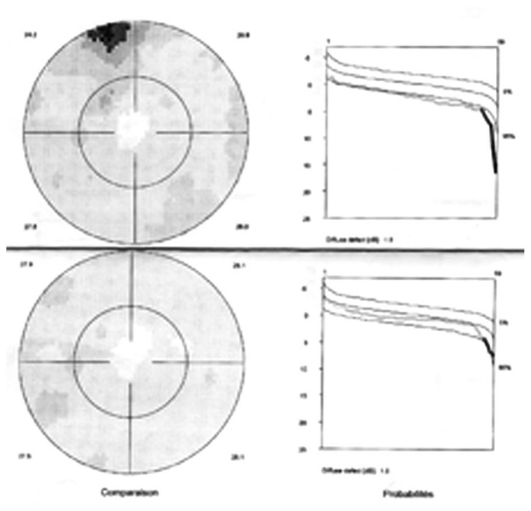
Octopus visual fields in MEWDS. Slight increase in mean defect (MD) and shallow relative scotoma at presentation (top image) with normalization 7 weeks later during the healing phase.

**Figure 4C:**
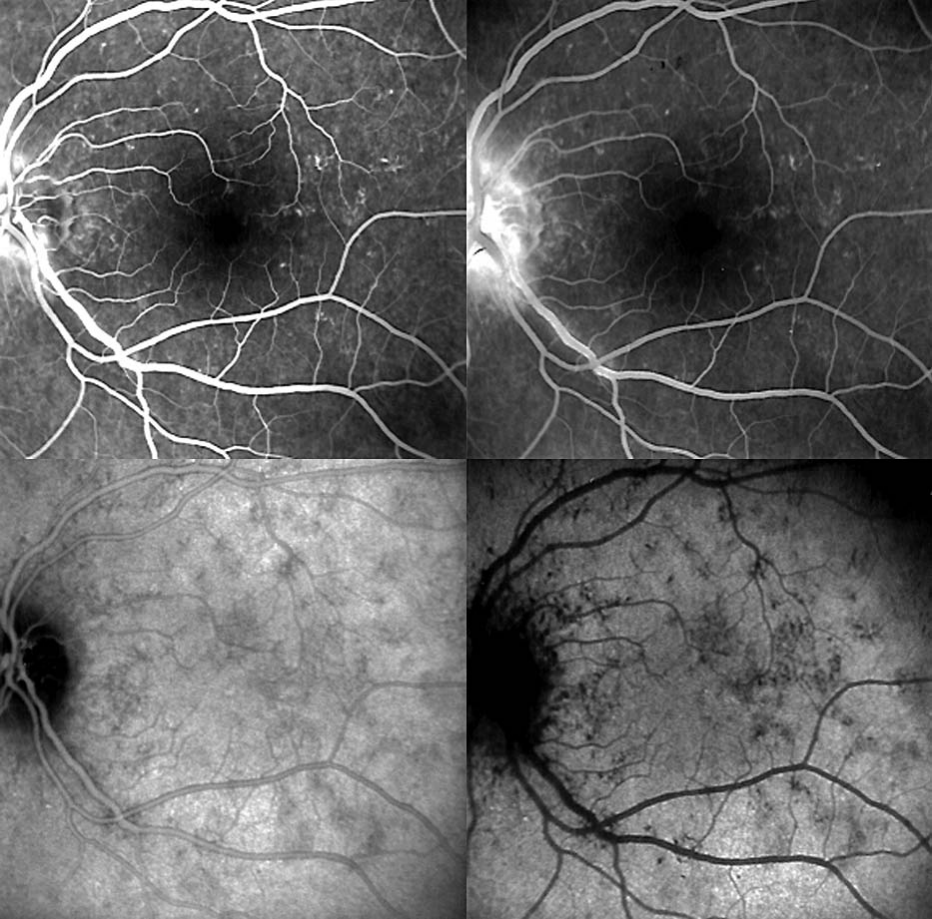
FA and ICGA frames in a patient with MEWDS. FA (top images) shows tiny hyperfluorescent spots in the posterior pole and disc leakage on the late frame (top right). ICGA (bottom images) shows extensive areas of choriocapillaris non- perfusion in the posterior pole, well visible in the intermediate phase (bottom left) but much clearly outlined in the late phase (bottom right).

**Figure 4D:**
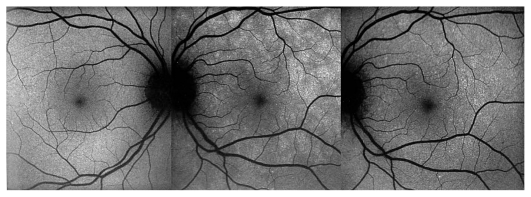
Fundus auto-fluorescence (FAF) in MEWDS. Geographic and confluent areas of increased FAF in the left diseased eye (middle picture) corresponding to dark ICGA areas which almost completely recovered in the healing stage (right image).
